# Weight bias among undergraduate women with health-related majors: a systematic review

**DOI:** 10.1186/s40337-025-01275-1

**Published:** 2025-06-12

**Authors:** Carly R. Pacanowski, Diane Vizthum, Sarah E. Katz, Christine Skubisz

**Affiliations:** 1 University of Delaware, Newark, DE USA; 2 Bentley University, Waltham, MA USA

## Abstract

**Background:**

Weight bias in healthcare has negative consequences for both patients and providers. While weight bias has been examined among healthcare professionals, understanding levels of bias during education may aid in understanding when bias is most salient and allow for targeted intervention to reduce bias. The objective of this systematic review was to describe the levels of explicit, internalized, and implicit weight bias among undergraduate women majoring in health-related fields.

**Methods:**

This review was pre-registered with PROSPERO (CRD42023478263). A systematic literature search was conducted using PubMed, APA PsycINFO (Proquest), CINAHL Plus with Full Text, Web of Science, and Cochrane Library (CENTRAL). Search terms centered on weight bias and undergraduate students studying health-related topics. Original research studies available in English that presented data on weight bias outcomes and presented data in the publication or provided data after being contacted for undergraduate women in health-related majors were included. Study quality was assessed with the Joanna Briggs Institute critical appraisal tool. Mean scores for quantitative scales and themes from qualitative studies were extracted and summarized.

**Results:**

The search produced 681 results; 14 studies were found eligible representing 3,260 women from eight countries. Eight different quantitative scales were used across 12 studies to assess aspects of weight bias; one assessed implicit weight bias and one subscale assessed internalized weight bias. Two of the 14 studies used qualitative methods. Most studies reported low or moderate weight bias but most scale developers did not include cut points. Scales relating to controllability of obesity or blame had scores most indicative of weight bias.

**Discussion:**

Undergraduate women in health-related majors have levels of explicit weight bias similar to levels that have been reported in healthcare professionals. There have been limited studies assessing internalized weight bias, implicit weight bias, and qualitative assessments in undergraduate women majoring in health-related fields.

**Supplementary Information:**

The online version contains supplementary material available at 10.1186/s40337-025-01275-1.

## Plain language summary

Weight bias, or having negative thoughts toward and treating people at higher weights poorly, is common in healthcare and has harmful effects for both patients and those providing care. Researchers have collected and summarized data from studies on the levels of weight bias in healthcare professionals. We know less about the levels of weight bias among students studying health-related fields who will later become healthcare professionals. Women are especially impacted by weight bias. It is important to understand the levels of weight bias in women studying health-related topics because then we can reduce weight bias during education and before women become healthcare professionals. The authors reviewed 14 published studies and found that weight bias exists in undergraduate women studying health-related majors. Future studies should track levels of weight bias as women proceed through college and evaluate the impact of how educational curricula may impact weight bias.

**Supplementary Information:**

The online version contains supplementary material available at 10.1186/s40337-025-01275-1.

## Introduction

Weight bias refers to negative attitudes and beliefs about individuals who have or are perceived to have high body weights [1]. Weight bias can be categorized as explicit, internalized, or implicit. Explicit weight bias consists of consciously held negative beliefs about individuals with high body weights [1]. Internalized weight bias is a type of explicit bias that occurs when individuals apply negative stereotypes about weight to themselves [2]. In contrast to explicit bias, implicit bias consists of subconscious beliefs and associations an individual may not be aware they hold [1]. Weight bias is one aspect of weight stigmatization, which is social devaluing and prejudice against individuals with high body weights.

In the healthcare setting, both patients and providers can be impacted by weight bias and stigma. Weight bias has been documented in healthcare professionals [3, 4] and trainees [5–7]. Healthcare professionals may dislike treating individuals living in larger bodies, believe they are non-compliant, or make insensitive comments on purpose or invertedly [5]. Healthcare professionals may spend less time with patients at higher body weights and engage in less patient-centered care [1]. For patients, experiencing weight stigma may result in delaying or avoiding care [8], and can be associated with worsened mental health [9] and using food as a coping mechanism. [10]

Healthcare professionals themselves may also be victims of weight bias and weight stigmatization. If patients have a preference, they are more likely to prefer a physician who is not of higher weight [11]. Patients report higher confidence in physicians who do not have obesity [12] and are more inclined to follow their advice. [13]

The vast majority of nurses [14] and allied health professionals [15–18] and a substantial portion of physicians [19] are women. Women are particularly vulnerable to weight stigma and tend to experience more weight stigma compared to men [20]. Women may be more likely to engage in unhealthy coping mechanisms as a result of stigma. For example, women have been found to exercise less after experiencing weight stigma in comparison to men [21], and women who are overweight eat more in response to viewing weight-stigmatizing content compared to neutral content [22]. Women are also more likely to internalize weight bias, and internalized weight bias is associated with higher levels of disordered eating, depression, and visceral fat [23, 24].

Reduction of weight bias and prevention of weight bias internalization is crucial for improved health of providers, particularly women, and the patients they care for. While previous systematic reviews have assessed weight bias in healthcare professionals [3, 4], to the best of our knowledge, data on levels of weight bias in undergraduate women majoring in health-related fields have not been presented in a systematic review. A comprehensive understanding of the types and degree of weight bias present early in the healthcare education trajectory can guide the development of programs to target and reduce weight bias among future healthcare professionals at the most appropriate time during training. The objective of this systematic review was to describe the levels of explicit, internalized, and implicit weight bias among undergraduate women majoring in health-related fields.

## Methods

This systematic review was conducted according to the PRISMA guidelines [25] and was pre-registered with PROSPERO (CRD42023478263). One amendment was made after the protocol was registered to clarify the research question.

### Search strategy

A health science librarian (S.E.K.) searched the following databases through September 30, 2023: PubMed, APA PsycINFO (Proquest), CINAHL Plus with Full Text, Web of Science, and Cochrane Library (CENTRAL) using terms related to weight bias, weight stigma, weight prejudice, health science students, nutrition students, and dietetic students. The search was developed in PubMed and modified to fit the parameters of the other databases. There were no additional limits placed on date, language, or article type. Full search strategies for each database can be viewed in supplementary Table S1.

### Eligibility criteria

Articles were included if they: were an original research study published in a peer-reviewed academic journal; were available in the English language; included participants attending a college or university as an undergraduate student with a health-related major at the time of data collection; included data from females/women* that could be examined separately from males/men; and reported outcomes include weight bias, fat bias, weight stigma, views on obesity, or implicit or explicit weight bias/stigma. Health-related majors were defined broadly to include any majors that involve studying to be a professional in physical or mental health (e.g. medicine, nursing, allied health, psychology) or work in an alternate health-related occupation (e.g. government, community setting). Authors were contacted if their study contained data that was not reported in a way that met inclusion criteria (i.e. if the paper reported data for a mixed sample of men and women), and the needed data was requested. Studies that did not meet inclusion criteria or were not able to provide data that met inclusion criteria were excluded.

### Study selection

Duplicate search results were removed using Zotero. All article titles and abstracts were independently screened by 3 research assistants for inclusion of data reports in the full text review. Any disagreements were resolved by discussion with the full research team. All full text reports were independently screened by 3 research assistants for inclusion in the review. Any disagreements were resolved by discussion with the full research team.

### Data collection process

For each eligible study, two reviewers independently extracted data. Extracted data was compared and discrepancies were resolved by discussion. The following data were extracted from studies as available: study characteristics (authors, publication year, study purpose, research question, and hypothesis, study location and country, study design), participant characteristics (inclusion and exclusion criteria, sample size, number or percentage of females/women, race/ethnicity, age, and weight or Body Mass Index), means and standard deviations and/or qualitative themes for outcomes related to weight bias, weight stigma, or attitudes towards obesity. If a study tested an intervention aimed at changing weight bias or stigma, only pre-test values were recorded. For studies in which authors provided additional data, the means and standard deviations for desired scales for females/women health majors were computed using SPSS. Results were organized by type of weight bias assessed: explicit, internalized, or implicit.

### Quality assessment

The Joanna Briggs Institute (JBI) critical appraisal tools were used to assess study quality [26]. Each study was independently appraised by 2 research assistants. The final sample of studies included projects with three types of designs: cross-sectional studies, quasi-experimental studies (from which we used baseline data only), and qualitative studies. The analytical cross-sectional studies appraisal tool [27] was used to evaluate cross-sectional studies and baseline data from quasi-experimental studies, and the qualitative tool [28] was used to assess qualitative studies. Data were assessed at the outcome level. Characteristics assessed for quantitative outcomes included descriptions of inclusion criteria, study participants and setting, use of objective, standard criteria for measurement, valid and reliable measurement of outcomes, and identification of any confounding factors. Characteristics assessed for qualitative outcomes included congruency between the stated philosophical perspective, research methodology, study methods, data interpretation, and interpretation of results. The three possible outcomes for the JBI quality assessment were “include, exclude, or seek additional information.”

## Results

### Study selection

**Fig. 1:**
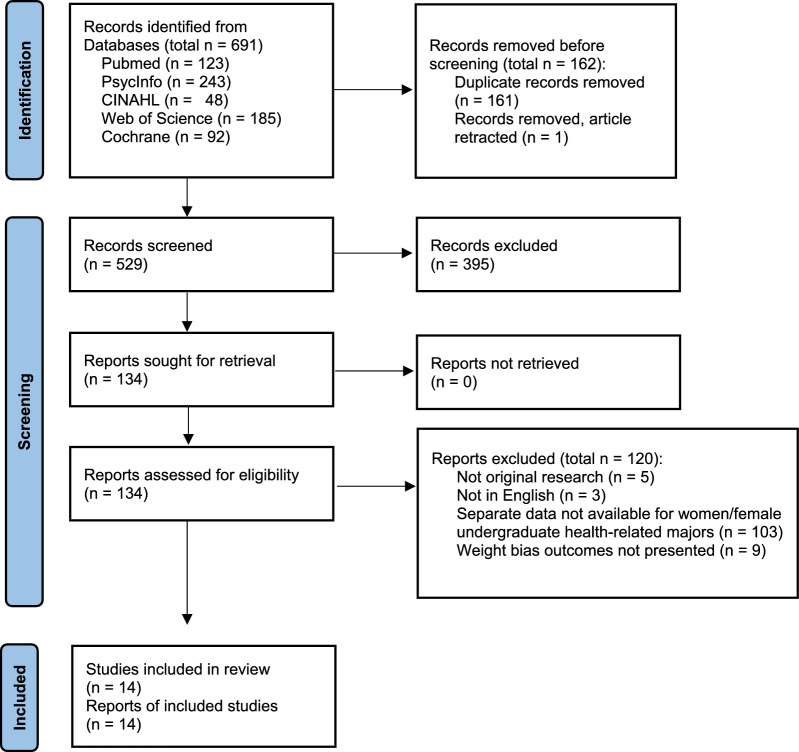
PRISMA Diagram

The initial search produced 681 results. After the removal of duplicates and one study that had been retracted, 529 records were screened, and 134 reports were retrieved for full text screening. The majority of the studies not sought for retrieval did not have undergraduate participants. Of the 134 reports, 9 were found to be eligible. Authors were contacted for an additional 16 articles that had the necessary data but did not report it (i.e. reported data for a mixed sample of men and women); of these 5 data sets were retrieved and able to be used, resulting in 14 studies ultimately included [29–42]. Reports were most commonly ineligible due to having data that was not available for females/women only (k = 68) (e.g [43].). Figure 1 displays the PRISMA diagram with full details of the selection process.

### Quality assessment

All 14 studies included in the final analysis were determined to be eligible for inclusion based on the Joanna Briggs Institute criteria. There were no discrepancies between reviewers on Joanna Briggs Institute critical appraisal ratings. Refer to supplementary Tables S2 and S3 for item-level assessments of included studies.

### Study characteristics

Characteristics of the 14 included studies are found in Table 1. Studies had data from participants in the United States (k = 5), Turkey (k = 3), Canada (k = 2), Hong Kong (k = 2) and one study each for Brazil, Spain, Taiwan, and Mexico. Among the possible health-related majors, nursing students were most commonly represented (k = 5), followed by nutrition/dietetics (k = 3), exercise science (k = 3), and one each for psychology, health, and other health majors. Thirteen studies employed a cross-sectional design and 1 used a quasi-experimental approach. Twelve studies measured weight bias quantitatively and 2 measured it qualitatively. Of 8 different quantitative measures of weight bias, 6 assessed explicit weight bias: the Fat Phobia Scale (FPS, k = 3), Attitudes Toward Obese Persons (ATOP, k = 3), Anti Fat Attitudes Test (AFAT, k = 3), Anti Fat Attitudes questionnaire (AFA, k = 1), GAMS-27 obesity prejudice scale (k = 1), and the Stigma scale (k = 1). One study assessed internalized weight bias using the fear of fat subscale of the Anti Fat Attitudes Questionnaire (AFA) and 1 study assessed implicit weight bias using the Implicit Association Test [42, 44]. Two qualitative studies used semi-structured interview guides to explore the experiences of nutrition and dietetics students in Canada.

**Table 1:** Study Characteristics

First Author, Year	Sample Size	Country	Participant Major	Study Design	Weight Bias Assessment Tool (8 total)	Bias Type	Joanna Briggs Institute Critical Appraisal^b^
Langdon [30]	145	USA	Exercise Science	Cross-sectional	Fat Phobia Scale (FPS) (1) Anti Fat Attitudes Test (AFAT) (2) *Subscales:* Social/Character Disparagement Physical/Romantic Unattractiveness Weight Control/Blame	Explicit	include
Chambliss [31]	57	USA	Exercise Science	Cross-sectional	Anti Fat Attitudes Test (AFAT) (2) *Subscales:* Social/Character Disparagement Physical/Romantic Unattractiveness Weight Control/Blame	Explicit	include
Darling [32]	212	USA	Nursing	Cross-sectional	Attitudes Towards Obese Persons (ATOP) (3) Beliefs About Obese Persons scale (BAOP) (4)	Explicit	include
Ciao [33]	50	USA	Psychology	Quasi-experimental	Anti Fat Attitudes Test (AFAT) (2) *Subscales:* Social/Character Disparagement Physical/Romantic Unattractiveness Weight Control/Blame	Explicit	include
Alameda [29]	45	USA and Mexico	Physical Education and Exercise Science	Cross-sectional	Implicit Association Test: Weight Bias (5)	Implicit	include
Ozaydin [34]	205	Turkey	Nursing	Cross-sectional	GAMS-27 obesity prejudice scale (6) Stigma scale (7)	Explicit	include
Usta [35]	525	Turkey	Nursing	Cross-sectional	Fat Phobia Scale (1) Attitudes Towards Obese Persons (ATOP) scale^a^ (3) Beliefs about Obese Persons (BAOP) scale^a^ (4)	Explicit	include
Yildiz [37]	489	Turkey	Health	Cross-sectional	GAMS-27 obesity prejudice scale (6) Stigma scale (7)	Explicit	include
Dwyer [38]	20	Canada	Nutrition/Dietetics	Cross-sectional	Qualitative interview	Explicit	include
Bessey [39]	10	Canada	Nutrition/Dietetics	Cross-sectional	Qualitative interview	Explicit, Internalized	include
Poon [40]	306	Hong Kong	Nursing	Cross-sectional	Fat Phobia Scale (FPS) (1)	Explicit	include
Tsai [36]	141	Hong Kong and Taiwan	Varied health majors	Cross-sectional	Attitudes Towards Obese Persons (ATOP) scale (3) Beliefs about Obese Persons (BAOP) scale (4)	Explicit	include
Alvarenga [41]	588	Brazil	Nutrition/Dietetics	Cross-sectional	Anti Fat Attitudes Test (ATAT) (2)	Explicit	include
Rodriguez-Gazquez [42]	467	Spain	Nursing	Cross-sectional	Anti Fat Attitudes (AFA) questionnaire (8) *Subscales:* Dislike Willpower Fear of Fat	Explicit, Internalized	include

^a^Turkish version

^b^All studies were assessed using the Joanna Briggs Institute Analytical Cross-Sectional Study checklist

## Weight bias outcomes

Weight bias outcomes are presented in Table 2.

**Table 2:** Weight Bias Results

Scale	Author/Year	Population	Results (Mean (SD))	Scale details (number of items, rating scale, scoring range, interpretation, and sample item(s))	Notes/Author Interpretation	Scale developers’ cut point
Explicit Bias						
*Total/Overall/General Measures*						
Anti Fat Attitudes questionnaire (AFA) – total, Spanish Adaptation 1994	Rodriguez-Gazquez 2020	Nursing, Spain	27.36^a^	13 items, 7-point Likert scale, scores range from 12–84 (higher scores indicate greater antifat attitudes) 3 subscales (American version uses a 0–9-point Likert scale) *See subscales for sample items*	Used 12 of 13 items (item no. 2 dropped to improve Cronbach’s alpha)	Not indicated
Antifat Attitudes Test (AFAT) – total 1997	Chambliss 2004	Exercise science, USA	2.20 (0.58)	47 items, 5- point Likert scale, standardized scores range from 1–5 (greater antifat bias) 3 subscales and ‘additional items’ *See subscales for sample items*	Authors state mean score > 3.0 indicates antifat bias	Not indicated
	Alvarenga 2022	Nutrition, Brazil	1.76^b^			
	Ciao 2011	Psychology, USA	2.01 (0.48)			
Fat Phobia Scale (FPS) 1984	Usta 2021	Nursing, Turkey	3.78 (0.52)	Participants are asked to rank which of a pair of adjectives is closest to their feelings about obese people scale of 1–5 (greater fatphobia) *Examples:* *Active/inactive* *Strong/weak* *Lazy/industrious*	Authors describe as “moderate levels.”	3.6 = average fatphobia 4.4 or higher = high fatphobia
	Langdon 2016	Exercise science/health science, USA	3.57 (0.82)		Authors describe as “midrange”, significantly different from midpoint of the scale	3.6 = average fatphobia 4.4 or higher = high fatphobia
	Poon 2008	Nursing, Hong Kong	3.48 (0.44)			
Attitudes Towards Obese Persons (ATOP) scale 1991	Darling 2019	Nursing, USA	67.67 (14.98)	20 items, 6-point Likert scale, scores range from 0–120 (more positive attitude towards people with obesity) *Obese workers cannot be as successful as other workers* *Obese people are just as healthy as nonobese people*		
	Tsai 2019	Varied health majors, Hong Kong and Taiwan	74.08 (11.25)			
	Usta 2021* Turkish version	Nursing, Turkey	60.07 (0.67)		Authors describe as “moderate levels” given the lack of cutoff scores	Not indicated
GAMS-27 obesity prejudice scale 2015	Ozaydin 2022	Nursing, Turkey	75.94 (9.93)	27-item scale, scores range from 27–135 points(more prejudice) 68 or lower = unprejudiced 68.01–84.99 = inclined to prejudice 85 or higher = prejudiced *Not available*	*Scoring information obtained from authors, original paper is in Turkish*	Original article containing cut points in Turkish
	Yildiz 2019	Health science, Turkey	74.24 (10.46)			
Stigma scale 2013	Ozaydin 2022	Nursing, Turkey	47.62 (9.51)	Scores range from 22–110 > 55 = high stigmatization tendency *Not available*	*Scoring information obtained from authors, original paper is in Turkish*	Original article containing cut points in Turkish
*Subscales relating to dislike and disparagement*						
Anti Fat Attitudes questionnaire (AFA), Spanish Adaptation dislike subscale	Rodriguez-Gazquez 2020	Nursing, Spain	7.606^a^	7 items, 7-point Likert scale, scores range from 6–42(more antifat attitudes) *I really don’t like fat people much*	Used 6 items of 7-item scale (item no. 2 dropped to improve Cronbach’s alpha)	Not indicated
Anti Fat Attitudes Test (AFAT) social/character disparagement subscale	Langdon 2016	Exercise science/health science, USA	1.91 (0.58)	15 items, 5-point Likert scale, scores range from 1–5 (greater antifat bias) *I’d lose respect for a friend who started getting fat* *Most fat people don’t keep their surroundings neat and clean*	Authors describe as “midrange”, significantly different from midpoint of the scale	Not indicated
	Chambliss 2004	Exercise science, USA	1.69 (0.58)		Authors state mean score > 3.0 indicates antifat bias	Not indicated
	Ciao 2011	Psychology, USA	1.50 (0.45)			
Anti Fat Attitudes Test (AFAT) physical/romantic unattractiveness subscale	Langdon 2016	Exercise science/health science, USA	2.68 (0.64)	10 items, 5-point Likert scale, scores range from 1–5 (greater antifat bias) *I would not want to continue in a romantic relationship if my partner became fat* *If I were single, I would date a fat person*	Authors describe as “midrange”, significantly different from midpoint of the scale	Not indicated
	Chambliss 2004	Exercise science, USA	2.75 (0.69)		Authors state mean score > 3.0 indicates antifat bias	Not indicated
	Ciao 2011	Psychology, USA	2.50 (0.50)			
Scale	Author/Year	Population	Results (Mean (SD))	Scale details (number of items, rating scale, scoring range, interpretation, and sample item(s))	Notes/Author Interpretation	Scale developers’ cut point
*Scales and subscales relating to controllability and blame*						
Beliefs About Obese Persons (BAOP) scale 1991	Darling 2019	Nursing, USA	17.09 (5.28)	8 items, 6-point Likert scale, scores range from 0–48 (greater belief that obesity is not controllable) *Obesity often occurs when eating is used as a form of compensation for lack of love or* *attention* *Obesity is rarely caused by a lack of willpower* *Statements ranked on a 6-point Likert scale from strongly disagree to strongly agree*		
	Tsai 2019	Varied health majors, Hong Kong and Taiwan	20.00 (5.71)			
	Usta 2021* Turkish version	Nursing, Turkey	23.29 (0.80)		Authors describe as “moderate levels” given the lack of cutoff scores, state participants believe “obesity is controlled by the individual.”	Not indicated
Anti Fat Attitudes questionnaire (AFA), Spanish Adaptation willpower subscale	Rodriguez-Gazquez 2020	Nursing, Spain	11.01^a^	3 items, 7-point Likert scale, scores range from 7–21(higher antifat attitudes) *Some people are fat because they have no willpower*		Not indicated
Anti Fat Attitudes Test (AFAT) weight control/blame subscale	Langdon 2016	Exercise science/health science, USA	2.86 (0.65)	9 items, 5-point Likert scale, scores range from 1–5(greater antifat bias) *The idea that genetics causes people to be fat is just an excuse* *Fat people have no willpower*	Authors describe as “midrange”	Not indicated
	Chambliss 2004	Exercise science, USA	2.72 (0.68)		Authors state mean score > 3.0 indicates antifat bias	Not indicated
	Ciao 2011	Psychology, USA	2.53 (0.71)			
Scale	Author/Year	Population	Results (Mean (SD))	Scale details (number of items, rating scale, scoring range, interpretation, and sample item(s))	Notes/Author Interpretation	Scale developers’ cut point
Internalized Weight Bias						
Anti Fat Attitudes questionnaire (AFA), Spanish Adaptation fear of fat subscale	Rodriguez-Gazquez 2020	Nursing, Spain	8.75^a^	3 items, 7-point Likert scale, 3–21(higher antifat attitudes) *I feel disgusted with myself when I gain weight*		Not indicated
Implicit Weight Bias						
Implicit Association Test: Weight Bias 1995 Good vs. bad Smart vs. stupid Motivated vs. lazy	Alameda 2015	Physical education and exercise, USA and Mexico	Good vs bad: US: 4.45 (2.84)* Mexican: 2.75 (3.38)* Smart vs stupid: US: 3.78 (2.97)* Mexican: 6.69 (4.12)* Motivated vs lazy: US: 4.56 (3.60)* Mexican: −0.10 (5.36)	Scores different from 0 = antifat bias (denoted by *) Score is calculated based on the number of words correctly classified with the adjectives (e.g. good/bad) when they are paired with “fat people” vs “thin people” *“Fat people” or “thin people” paired with an adjective (good/bad)* *Words to classify include obese, sluggish, slim, eager, large*		

Qualitative		Themes *Illustrative Quotes*
Dwyer 2016	Nutrition/Dietetics, Canada	Increased knowledge of obesity and understanding of complexity through curriculum Increased empathy and ability to avoid stereotypes *“We learned all about the background of obesity and all the factors that contribute to it [in clinical nutrition course], so now I don’t just see someone who’s overweight and assume they have bad habits.”*
Bessey 2021	Nutrition/Dietetics, Canada	Weight stigma in dietetics education Doing dietitian (presenting self as a “good dietitian”) Questioning weight stigma *“And she said she does that too. Before she ever does interviews. She tries to lose a bunch of weight and she recommended that I do the same thing because we’re judged on that.”*

^a^Standard deviation not reported for female-specific data

^b^Study reported Median (IQR) of 60 (53; 69), standardized score was calculated by dividing the total score by the number of items for ease of comparison with other studies

### Explicit bias: overall measures

Six scales assessed overall weight bias. The Fat Phobia Scale and GAMS-27 obesity prejudice scale provide cutoffs for assessing scores. The Fat Phobia Scale (FPS) mean scores ranged from 3.48 to 3.78, scores of 3.6–4.4 are considered moderate fat phobia [45]. The mean GAMS-27 obesity prejudice scores of 74.24 and 75.94 fell into the intermediate “inclined to prejudice” classification [37]. Other scales do not provide cutoff scores for classification. When compared to the theoretical midpoint of the scale, The Anti Fat Attitudes questionnaire (AFA), Anti Fat Attitudes Test (AFAT), and Attitudes Toward Obese Persons (ATOP) [46] scale scores were all on the side of the scale indicating lower weight bias; however, further interpretation is not possible.

### Studies measuring subscales related to explicit weight bias

Subscales assess specific weight bias constructs, including dislike, social/character disparagement, physical/romantic unattractiveness, and beliefs about controllability of weight. The Ant Fat Attitudes Test (AFAT) subscales have possible scores of 1–5. Lower scores were reported for social/character disparagement (means 1.50–1.91) compared to physical/romantic unattractiveness (means 2.50–2.68) and weight control/blame (means 2.53–2.86). The Anti Fat Attitudes questionnaire (AFA) similarly had lower scores for dislike 7.60 (out of possible 42) compared to willpower 11.01 (out of possible 21). Weight control or blame was also assessed with the Beliefs About Obese Persons (BAOP) scale [46]. This scale assesses beliefs about the controllability of obesity and is scored from 0–48, with higher scores indicating greater belief that obesity is not controllable (and therefore not the fault of the individual). The Beliefs About Obese Persons (BAOP) mean scores of 17.06–23.39 indicated a tendency towards the belief that weight is controllable.

### Studies measuring internalized weight bias

One study of nursing students in Spain found a score of 8.75 on the Anti Fat Attitudes questionnaire (AFA) fear of fat subscale (possible scores of 3–21). [42]

### Studies measuring Implicit weight bias

One study measured implicit weight bases using the weight bias version of the Implicit Association Test and found implicit weight bias in students who were studying physical education and exercise science in the United States and Mexico. [29]

### Studies with qualitative measures

Two studies used qualitative measures with samples of nutrition/dietetics students in Canada. One assessed whether students felt the nutrition curriculum influenced their attitudes towards people with obesity. Four themes emerged: increased knowledge of obesity, understanding the complexity of obesity, increased empathy, and better ability to avoid negative stereotypes [38]. Overall, participants felt their weight bias decreased as a result of their education. In contrast, a study of nutrition/dietetics students in Canada explored the experience of weight stigma in nutrition/dietetics students in larger bodies [39]. Themes included the presence of weight stigma throughout their education, the pressure to “perform” as a dietitian, and resistance to weight stigma. These students felt stigmatized during their professional training and were concerned about being stigmatized in the future as healthcare providers.

## Discussion

Fourteen studies were systematically identified and reviewed with data on weight bias available from undergraduate women studying a health-related field. In total, 3,260 women were represented in these studies as participants. Of the 14 studies, only 1 study assessed internalized weight bias, and only 1 study assessed implicit weight bias. Out of 8 different scales that quantitatively measured weight bias (7 scales measured explicit weight bias and 1 scale measured implicit weight bias), only one subscale (the Anti Fat Attitude (AFA) questionnaire fear of fat subscale) measured internalized weight bias. The majority of studies reported that participants exhibited low or moderate weight bias. Zero studies reported a weight bias score indicative of high weight bias (identified by author suggestion or scale cut point), which is encouraging. However, while researchers who developed the Fat Phobia Scale (FPS), GAMS-27 obesity prejudice scale, and Stigma scale provided cut points, researchers who developed the Anti Fat Attitudes questionnaire (AFA), Anti Fat Attitudes Test (AFAT), Attitudes Toward Obese Persons (ATOP) and Beliefs About Obese Persons (BAOP) did not provide cut points. Authors of included studies who made interpretations of results often used the scale’s midpoint to do so. Scales relating to controllability of obesity or blame had scores most indicative of weight bias.

The scores on quantitative measures of weight bias in undergraduate women studying health-related majors were similar to scores reported elsewhere for health professionals. For example, a systematic review and meta-analysis that assessed weight bias among healthcare professionals found a pooled mean Fat Phobia Scale (FPS) score of 3.48 (SE = 0.05) on a scale of 1–5, Attitudes Toward Obese People (ATOP) pooled mean score of 69.30 (SE = 1.77) on a scale of 0–120 [3]. The results reported in the present review of undergraduate women were that mean Fat Phobia Scale (FPS) scores ranged from 3.48–3.78, mean Attitudes Toward Obese Persons (ATOP)_scores ranged from 60.07 to 72.80. Given that healthcare professionals’ weight bias negatively impacts patients [1], and levels of weight bias among undergraduate students are similar to that of healthcare professionals, providing education about weight bias as part of the pre-health professional curriculum could be a reasonable intervention.

Education targeting information about the controllability of obesity may be most impactful. Undergraduate students'scores on the Beliefs About Obese Persons (BAOP) scale were on the side of the midpoint indicating a belief that obesity is controllable (higher scores indicate belief that obesity is not controllable). The Beliefs About Obese Persons (BAOP) scale was the only scale with scores falling on the side of the midpoint indicating higher weight bias. Several studies have found a relationship between receiving education on the genetic and environmental causes of obesity and decreased levels of implicit weight bias. Health students who ranked their education on the genetic causes of obesity as “excellent” [47] or who received an intervention on the genetic and environmental causes of obesity [48] had lower implicit bias compared to their peers. Other studies have shown that nutrition and medical education is associated with lower weight bias. For example, fourth year nutrition and nursing students in the UK had lower weight bias than first year nutrition students [49]. In Brazil, registered dietitians and nutrition students had lower scores on the weight control/blame subscale of the Antifat Attitudes Test (AFAT) compared to lay people [50]. These findings are consistent with one of the qualitative studies included in the present review which indicated that education has the potential to increase understanding of the complexity of obesity and reduce bias. [38]

The current review had several strengths, including the rigor of the data collection. The review was pre-registered with PROSPERO, followed the PRISMA guidelines, and the search was conducted by a health science librarian. Two, and in some stages 3, research assistants independently screened studies, extracted data, and performed a quality assessment of each study. This is the first systematic to report research on levels of weight bias in undergraduate women studying in health-related majors, a population whose levels of weight bias may impact future patient care, and who are personally at risk for adverse effects of internalized weight bias.

Limitations of the present review should be considered. Several studies that were reviewed for inclusion did not include the data necessary for analysis. Authors were contacted to request this data, however the percentage authors who were able and willing to provide the necessary additional data was low (~ 31%) and it is possible that levels of weight bias differ in the studies that were not able to be included. Studies that were not available in English were not included. While the included studies represented a range of countries, it is possible that studies that were translated into English were different in some way from studies that were not available in English and therefore excluded. Cross-cultural comparisons were not possible due to the heterogeneity of scales used in studies from different countries and the small sample size. Considering how weight bias exists culturally is an important area for future research, as body image varies between countries and may impact weight bias and attitudes about weight, as highlighted in a 2024 systematic review [51]. Finally, the publication year of studies included ranged from 2004 to 2022, with an average publication year of 2016.6 and standard deviation of 5.4 years. It is possible that levels of weight bias among college students have changed over time and more recent studies are needed to assess this possibility.

Multiple future directions exist based on the results of this systematic review. In this project, 13 of the 14 included studies used a cross sectional design. Future research could use a longitudinal approach to follow students throughout their education and examine the effect of curriculum on weight bias. The way in which weight is covered in health-related programs can impact students’ weight bias. For example, a longitudinal study has been conducted with medical school students and found those with more training in dealing with “difficult patients” (to include those with obesity) had higher implicit bias [52]. Other retrospective data found final year students who stated their education on the genetic/environmental causes of obesity was “excellent” had lower implicit bias and those who said their education was on this issue “poor” had higher explicit weight bias [47]. Initial studies have found weight bias reduction interventions to be feasible to implement and valuable to students and faculty. [43, 48, 53]

As weight stigma has come to the forefront of research in the last decade, it is notable that 6 of the 8 quantitative measures represented in the final set of included studies were published in a short time span between 1984 and 1997. Two measures were published much later in 2015 (GAMS-27 obesity prejudice scale) and 2022 (Stigma scale). Psychometric properties of weight stigma measures have been examined, however, an analysis of how weight stigma has been measured over time is also fruitful direction for future research. [54]

The type of weight bias (explicit, internalized, implicit) may influence outcomes both for future professionals and their patients. There may be different conceptual implications for women with health-related majors considering the type of weight bias. Young women are particularly impacted by internalized weight bias and the adverse physiological and psychological consequences have been well demonstrated [55]. The present review found that only 1 quantitative study assessed internalized weight bias and 1 quantitative study assessed implicit weight bias among women studying health-related topics. In the systematic review and meta-analysis of healthcare providers, the pooled level of implicit bias was reported to be moderate [56]. It is not possible to compare the level of implicit bias reported by studies presented in the review to the one study we found that measured implicit bias in students [44] in the present review due to the way the authors reported implicit bias (mean and standard deviation as compared to *d* score calculated for the Implicit Association Test.) [57] A qualitative study included in the present review found that students in dietetics perceive pressure to be thin to be a good dietitian [39]. Future studies assessing both internalized and implicit bias among women studying to become healthcare professionals are needed to elucidate whether implicit bias is also at a moderate level during undergraduate education or bias is higher or lower among students compared to healthcare professionals. Similar work could be done to investigate internalized bias among women studying health-related topics and whether internalized bias among students later impacts how professionals interact with patients. Healthcare providers and patients can both suffer from the adverse effects of weight bias and internalized weight bias has been found to be particularly harmful for women [58]. There is much work to be done in understanding weight bias before students transition to healthcare professionals, and the findings could direct where intervention efforts will be most fruitful. In conclusion, the results of this study identify that weight bias exists in undergraduate women studying health-related majors. Moreover, undergraduate women in health-related majors have levels of explicit weight bias similar to levels that have been reported by healthcare professionals. Given that weight bias exists prior to students entering their chosen professions, it is important to consider ways that weight bias can be reduced during formal education. 

## Supplementary Information

Supplementary Material 1.

Supplementary Material 2.

## Data Availability

No datasets were generated or analysed during the current study.

## References

[1] Phelan SM, Burgess DJ, Yeazel MW, Hellerstedt WL, Griffin JM, van Ryn M. Impact of weight bias and stigma on quality of care and outcomes for patients with obesity. Obes Rev. 2015;16(4):319–26. 10.1111/obr.12266.25752756 10.1111/obr.12266PMC4381543

[2] Pearl RL, Puhl RM. Weight bias internalization and health: a systematic review. Obes Rev. 2018;19(8):1141–63. 10.1111/obr.12701.29788533 10.1111/obr.12701PMC6103811

[3] Lawrence BJ, Kerr D, Pollard CM, et al. Weight bias among health care professionals: a systematic review and meta-analysis. Obesity. 2021;29(11):1802–12. 10.1002/oby.23266.34490738 10.1002/oby.23266

[4] Panza GA, Armstrong LE, Taylor BA, Puhl RM, Livingston J, Pescatello LS. Weight bias among exercise and nutrition professionals: a systematic review. Obes Rev Off J Int Assoc Study Obes. 2018;19(11):1492–503. 10.1111/obr.12743.10.1111/obr.1274330176183

[5] Puhl RM, Luedicke J, Grilo CM. Obesity bias in training: Attitudes, beliefs, and observations among advanced trainees in professional health disciplines. Obesity. 2014;22(4):1008–15. 10.1002/oby.20637.24124078 10.1002/oby.20637PMC3968226

[6] Swift JA, Hanlon S, El-Redy L, Puhl RM, Glazebrook C. Weight bias among UK trainee dietitians, doctors, nurses and nutritionists. J Hum Nutr Diet. 2013;26(4):395–402. 10.1111/jhn.12019.23171227 10.1111/jhn.12019

[7] Phelan SM, Dovidio JF, Puhl RM, et al. Implicit and explicit weight bias in a national sample of 4732 medical students: the medical student CHANGES study. Obesity. 2014;22(4):1201–8. 10.1002/oby.20687.24375989 10.1002/oby.20687PMC3968216

[8] Drury CAA, Louis M. Exploring the association between body weight, stigma of obesity, and health care avoidance. J Am Acad Nurse Pract. 2002;14(12):554–61. 10.1111/j.1745-7599.2002.tb00089.x.12567923 10.1111/j.1745-7599.2002.tb00089.x

[9] Emmer C, Bosnjak M, Mata J. The association between weight stigma and mental health: A meta-analysis. Obes Rev. 2020;21(1): e12935. 10.1111/obr.12935.31507062 10.1111/obr.12935

[10] Puhl RM, Brownell KD. Confronting and coping with weight stigma: an investigation of overweight and obese adults. Obesity. 2006;14(10):1802–15. 10.1038/oby.2006.208.17062811 10.1038/oby.2006.208

[11] Goldring MR, Persky S. Preferences for physician weight status among women with overweight. Obes Sci Pract. 2018;4(3):250–8. 10.1002/osp4.162.29951215 10.1002/osp4.162PMC6009989

[12] Hash RB, Munna RK, Vogel RL, Bason JJ. Does physician weight affect perception of health advice? Prev Med. 2003;36(1):41–4. 10.1006/pmed.2002.1124.12473423 10.1006/pmed.2002.1124

[13] Puhl RM, Gold JA, Luedicke J, DePierre JA. The effect of physicians’ body weight on patient attitudes: implications for physician selection, trust and adherence to medical advice 2005. Int J Obes. 2013;37(11):1415–21. 10.1038/ijo.2013.33.10.1038/ijo.2013.3323507996

[14] Smiley RA, Allgeyer RL, Shobo Y, et al. The 2022 national nursing workforce survey. J Nurs Regul. 2023;14(1):S1–90. 10.1016/S2155-8256(23)00047-9.37012978

[15] Rogers D. Report on the academy/commission on dietetic registration 2020 needs satisfaction survey. J Acad Nutr Diet. 2021;121(1):134–8. 10.1016/j.jand.2020.10.018.33342515 10.1016/j.jand.2020.10.018

[16] American Physical Therapy Association. APTA Physical Therapy Workforce Analysis. Published online December 2020.

[17] Ledgerd and World Federation of Occupational Therapists R, World Federation of Occupational Therapists. WFOT report: WFOT human resources project 2018 and 2020. *World Fed Occup Ther Bull*. 2020;76(2):69–74. 10.1080/14473828.2020.1821475

[18] A Demographic Snapshot of SLPs: Data highlight some key characteristics of ASHA’s SLP members. *ASHA Lead*. 2019; 24(7):32–32. 10.1044/leader.AAG.24072019.32

[19] 2022 Physician Specialty Data Report. Published online 2022.

[20] Puhl RM, Andreyeva T, Brownell KD. Perceptions of weight discrimination: prevalence and comparison to race and gender discrimination in America. Int J Obes. 2008;32(6):992–1000. 10.1038/ijo.2008.22.10.1038/ijo.2008.2218317471

[21] Sattler KM, Deane FP, Tapsell L, Kelly PJ. Gender differences in the relationship of weight-based stigmatisation with motivation to exercise and physical activity in overweight individuals. Health Psychol Open. 2018;5(1):2055102918759691. 10.1177/2055102918759691.29552349 10.1177/2055102918759691PMC5846936

[22] Schvey NA, Puhl RM, Brown KD. The impact of weight stigma on caloric consumption. Obesity. 2011;19(10):1957–62. 10.1038/oby.2011.204.21760636 10.1038/oby.2011.204

[23] Boswell RG, White MA. Gender differences in weight bias internalisation and eating pathology in overweight individuals. Adv Eat Disord. 2015;3(3):259–68. 10.1080/21662630.2015.1047881.27042387 10.1080/21662630.2015.1047881PMC4814168

[24] Women felt more stigma about abdominal fat than men, regardless of body weight. American Heart Association. Accessed June 25, 2024. http://newsroom.heart.org/news/women-felt-more-stigma-about-abdominal-fat-than-men-regardless-of-body-weight

[25] Page MJ, McKenzie JE, Bossuyt PM, et al. The PRISMA statement: an updated guideline for reporting systematic reviews. BMJ. 2020. 10.1136/bmj.n71.33782057 10.1136/bmj.n71PMC8005924

[26] JBI Critical Appraisal Tools | JBI. Accessed June 27, 2024. https://jbi.global/critical-appraisal-tools

[27] Moola S, Munn Z, Tufanaru C, Aromataris E, Sears K, Sfetcu R, Currie M, Qureshi R, Mattis P, Lisy K, Mu P-F. Chapter 7: Systematic reviews of etiology and risk. In: Aromataris E, Munn Z, ed. *JBI Manual for Evidence Synthesis*. JBI; 2020. https://synthesismanual.jbi.global

[28] Lockwood C, Munn Z, Porritt K. Qualitative research synthesis: methodological guidance for systematic reviewers utilizing meta-aggregation. Int J Evid Based Healthc. 2015;13(3):179–87. 10.1097/XEB.0000000000000062.26262565 10.1097/XEB.0000000000000062

[29] Alameda MW, Whitehead J. Comparing levels of anti-fat bias between American and Mexican athletes and undergraduate physical education and exercise science students. Phys Educ. 2015. 10.18666/TPE-2015-V72-I5-4668.

[30] Langdon J, Rukavina P, Greenleaf C. Predictors of obesity bias among exercise science students. Adv Physiol Educ. 2016;40(2):157–64. 10.1152/advan.00185.2015.27068990 10.1152/advan.00185.2015

[31] Chambliss HO, Finley CE, Blair SN. Attitudes toward obese individuals among exercise science students. Med Sci Sports Exerc. 2004;36(3):468–74. 10.1249/01.MSS.0000117115.94062.E4.15076789 10.1249/01.mss.0000117115.94062.e4

[32] Darling R, Atav AS. Attitudes toward obese people: a comparative study of nursing, education, and social work students. J Prof Nurs. 2019;35(2):138–46. 10.1016/j.profnurs.2018.07.009.30902406 10.1016/j.profnurs.2018.07.009

[33] Ciao AC, Latner JD. Reducing obesity stigma: the effectiveness of cognitive dissonance and social consensus interventions. Obesity. 2011;19(9):1768–74. 10.1038/oby.2011.106.21546926 10.1038/oby.2011.106

[34] Ozaydin T, Kaya Tuncbeden MM. An investigation of the prejudice and stigmatization levels of nursing students towards obese individuals. Arch Psychiatr Nurs. 2022;40:109–14. 10.1016/j.apnu.2022.06.002.36064233 10.1016/j.apnu.2022.06.002

[35] Usta E, Bayram S, Altınbaş AÖ. Perceptions of nursing students about individuals with obesity problems: belief, attitude, phobia. Perspect Psychiatr Care. 2021;57(2):777–85. 10.1111/ppc.12613.32892386 10.1111/ppc.12613

[36] Tsai MC, Strong C, Latner JD, et al. Attitudes toward and beliefs about obese persons across Hong Kong and Taiwan: wording effects and measurement invariance. Health Qual Life Outcomes. 2019;17(1):134. 10.1186/s12955-019-1198-6.31362763 10.1186/s12955-019-1198-6PMC6668070

[37] Yildiz M, Yalcinoz Baysal H. Prejudice against obesity in university students studying in health-related departments. Perspect Psychiatric Care. 2018. 10.1111/ppc.12314.10.1111/ppc.1231430033627

[38] Dwyer JJM, Starr A, Mills C, Haines J. Undergraduate, female, nutrition students’ perceptions of curricular influence on attitudes toward individuals with obesity. Can J Diet Pract Res. 2016;77(4):177–82. 10.3148/cjdpr-2016-010.27744732 10.3148/cjdpr-2016-010

[39] Bessey M, Brady J, Lordly D, Leighteizer V. “This is what you’re supposed to do”: weight stigma in dietetics education. Fat Stud. 2021;10(2):184–96. 10.1080/21604851.2020.1859078.

[40] Poon M, Tarrant M. Obesity: attitudes of undergraduate student nurses and registered nurses. J Clin Nurs. 2009;18(16):2355–65. 10.1111/j.1365-2702.2008.02709.x.19374692 10.1111/j.1365-2702.2008.02709.x

[41] Alvarenga MDS, Obara AA, Takeda GA, Ferreira-Vivolo SRG. Anti-fat attitudes of Nutrition undergraduates in Brazil toward individuals with obesity. Ciênc Saúde Coletiva. 2022;27(2):747–60. 10.1590/1413-81232022272.02342021.10.1590/1413-81232022272.0234202135137829

[42] Rodríguez-Gázquez MDLA, Ruiz-Iglesias A, González-López JR. Changes in anti-fat attitudes among undergraduate nursing students. Nurse Educ Today. 2020;95: 104584. 10.1016/j.nedt.2020.104584.33011617 10.1016/j.nedt.2020.104584

[43] Bolter ND, Sosna D, Arauzo M, George GL. Changing perspectives among pre-health undergraduates through a brief weight bias pedagogical intervention. Health Educ J. 2023;82(4):361–75. 10.1177/00178969231159960.

[44] Alameda MW, Whitehead J. Comparing levels of anti-fat bias between american and mexican athletes and undergraduate physical education and exercise science students. *Phys Educ*. 2015;72(5). Accessed March 6, 2025. https://js.sagamorepub.com/index.php/pe/article/view/4668

[45] Bacon J, Scheltema K, Robinson B. Fat phobia scale revisited: the short form. Int J Obes. 2001;25(2):252–7. 10.1038/sj.ijo.0801537.10.1038/sj.ijo.080153711410828

[46] Allison DB, Basile VC, Yuker HE. The measurement of attitudes toward and beliefs about obese persons. Int J Eat Disord. 1991;10(5):599–607.

[47] Robinson EL, Ball LE, Leveritt MD. Obesity bias among health and non-health students attending an Australian university and their perceived obesity education. J Nutr Educ Behav. 2014;46(5):390–5. 10.1016/j.jneb.2013.12.003.24502966 10.1016/j.jneb.2013.12.003

[48] O’Brien KS, Puhl RM, Latner JD, Mir AS, Hunter JA. Reducing anti-fat prejudice in preservice health students: a randomized trial. Obes Silver Spring Md. 2010;18(11):2138–44. 10.1038/oby.2010.79.10.1038/oby.2010.7920395952

[49] Swift JA, Hanlon S, El-Redy L, Puhl RM, Glazebrook C. Weight bias among UK trainee dietitians, doctors, nurses and nutritionists. J Hum Nutr Diet Off J Br Diet Assoc. 2013;26(4):395–402. 10.1111/jhn.12019.10.1111/jhn.1201923171227

[50] Cassiano GS, Carvalho-Ferreira JP, Buckland NJ, da Cunha DT. Do registered dietitians, nutrition students, and laypeople perceive individuals with obesity differently? Int J Environ Res Public Health. 2021;18(17):8925. 10.3390/ijerph18178925.34501514 10.3390/ijerph18178925PMC8431474

[51] Abdoli M, Scotto Rosato M, Desousa A, Cotrufo P. Cultural Differences in Body Image: A Systematic Review. Soc Sci. 2024;13(6):305. 10.3390/socsci13060305.

[52] Sm P, Rm P, Se B, et al. The mixed impact of medical school on medical students’ implicit and explicit weight bias. Med Educ. 2015. 10.1111/medu.12770.10.1111/medu.12770PMC475531826383070

[53] Raffoul A, Andrade L, Acton RB, et al. Acceptability of an online module addressing weight bias: perspectives and attitudes of undergraduate health students and instructors. Can J Diet Pract Res Publ Dietit Can Rev Can Prat Rech En Diet Une Publ Diet Can. 2023;84(1):43–8. 10.3148/cjdpr-2022-028.10.3148/cjdpr-2022-02836413414

[54] Papadopoulos S, de la Piedad GX, Brennan L. Evaluation of the psychometric properties of self-reported weight stigma measures: A systematic literature review. Obes Rev Off J Int Assoc Study Obes. 2021;22(8): e13267. 10.1111/obr.13267.10.1111/obr.1326734105229

[55] Foster T, Eaton M, Probst Y. The relationship between internalised weight bias and biopsychosocial outcomes in children and youth: a systematic review. J Eat Disord. 2024;12(1):38. 10.1186/s40337-023-00959-w.38491402 10.1186/s40337-023-00959-wPMC10941429

[56] Lawrence BJ, Kerr D, Pollard CM, et al. Weight bias among health care professionals: A systematic review and meta-analysis. Obes Silver Spring Md. 2021;29(11):1802–12. 10.1002/oby.23266.10.1002/oby.2326634490738

[57] Greenwald AG, Nosek BA, Banaji MR. Understanding and using the Implicit Association Test: I an improved scoring algorithm. J Pers Soc Psychol. 2003;85(2):197–216. 10.1037/0022-3514.85.2.197.12916565 10.1037/0022-3514.85.2.197

[58] Hughes AM, Flint SW, Clare K, et al. Demographic, socioeconomic and life-course risk factors for internalized weight stigma in adulthood: evidence from an English birth cohort study. Lancet Reg Health Eur. 2024. 10.1016/j.lanepe.2024.100895.38745988 10.1016/j.lanepe.2024.100895PMC11092882

