# Supplementary material for: Weight bias among undergraduate women with health-related majors: a systematic review
Source: J Eat Disord. 2025 Jun 12;13:108. doi: 10.1186/s40337-025-01275-1 (PMC12160116; doi:10.1186/s40337-025-01275-1)
Supplement: Supplementary file 1 — Supplementary Material 1. [file 40337_2025_1275_MOESM1_ESM.docx]

Supplementary Table S1. Search Strategy by Database.

| Database | Search Strategy |
| --- | --- |
| **Cochrane Library CENTRAL** | ID Search  #1 MeSH descriptor: [Students, Medical] explode all trees  #2 MeSH descriptor: [undefined] explode all trees  #3 MeSH descriptor: [Students, Pharmacy] explode all trees  #4 MeSH descriptor: [Students, Premedical] explode all trees  #5 MeSH descriptor: [Students, Public Health] explode all trees  #6 MeSH descriptor: [Students, Dental] explode all trees  #7 MeSH descriptor: [Students, Health Occupations] explode all trees  #8 MeSH descriptor: [Education, Nursing, Baccalaureate] explode all trees  #9 MeSH descriptor: [Education, Medical, Undergraduate] explode all trees  #10 #1 OR #2 OR #3 OR #4 OR #5 OR #6 OR #7 OR #8 OR #9  #11 (Health Professions Students OR Health science students OR nutrition students OR nutritional science students OR dietetic students OR nursing students OR Nutrition Major OR kinesiology students):ti,ab,kw  #12 #10 OR #11  #13 MeSH descriptor: [Weight Prejudice] explode all trees  #14 MeSH descriptor: [Attitude of Health Personnel] explode all trees  #15 MeSH descriptor: [Body Weight] explode all trees  #16 #14 AND #15  #17 (stigma):ti,ab,kw  #18 #15 AND #17  #19 (bias):ti,ab,kw  #20 #15 AND #19  #21 #13 OR #16 OR #18 OR #20  #22 (Weight bias OR weight stigma OR weight shame OR weight guilt OR weight prejudice OR Weight Based Discrimination OR Weight Discrimination OR obesity stigma OR obesity bias OR weight related bias OR weight stereotypes OR Weight-Based Attitudes OR fat bias OR fat stigma OR (weight AND implicit bias) OR (weight AND explicit bias) OR weight stigmatization):ti,ab,kw  #23 #21 OR #22  #24 #12 AND #23  **#25 Trials** |
| **CINAHL (Ebscohost)** | ( (MH "Students, Medical") OR (MH "Students, Nursing") OR (MH "Students, Pharmacy") OR (MH "Education, Premedical") OR (MH "Students, Dental") OR  (MH "Students, Health Occupations") OR (MH "Education, Nursing, Baccalaureate") OR (MH "Students, Nursing, Baccalaureate") OR (MH "Applied Kinesiology/ED") OR (MH "Students, Dietetics") OR Health Professions Students OR Health science students OR nutrition students OR nutritional science students OR dietetic students OR nursing students OR Nutrition Major ) AND ( (MH "Weight Bias") OR ((MH "Attitude of Health Personnel") AND (MH "Body Weight")) OR ((MH "Body Weight") AND (MH "Stigma")) OR ((MH "Body Weight") AND bias) OR Weight bias OR weight stigma OR weight shame OR weight guilt OR weight prejudice OR Weight Based Discrimination OR Weight Discrimination OR obesity stigma OR obesity bias OR weight related bias OR weight stereotypes OR Weight-Based Attitudes OR fat bias OR fat stigma OR (weight AND implicit bias) OR (weight AND explicit bias) OR weight stigmatization )  **Limit – Articles** |
| **APA PsycINFO (ProQuest)** | **(**MAINSUBJECT.EXACT("Medical Students") ORMAINSUBJECT.EXACT("Nursing Students") OR (MAINSUBJECT.EXACT("Nursing Education") AND MAINSUBJECT.EXACT("College Students")) OR (MAINSUBJECT.EXACT("Medical Education") AND MAINSUBJECT.EXACT("College Students")) OR Health Professions Students OR Health science students OR nutrition students OR nutritional science students OR dietetic students OR nursing students OR Nutrition Major) AND ((MAINSUBJECT.EXACT("Body Weight") AND MAINSUBJECT.EXACT("Stigma")) OR Weight bias OR weight stigma OR weight shame OR weight guilt OR weight prejudice OR Weight Based Discrimination OR Weight Discrimination OR obesity stigma OR obesity bias OR weight related bias OR weight stereotypes OR Weight-Based Attitudes OR fat bias OR fat stigma OR (weight AND implicit bias) OR (weight AND explicit bias) OR weight stigmatization)  **Limit – Articles** |
| **PubMed** | ("Students, Medical"[Mesh] OR "Students, Nursing"[Mesh] OR "Students, Pharmacy"[Mesh] OR "Students, Premedical"[Mesh] OR "Students, Public Health"[Mesh] OR "Students, Dental"[Mesh] OR  "Students, Health Occupations"[Mesh] OR "Education, Nursing, Baccalaureate"[Mesh] OR "Kinesiology, Applied/education"[Mesh] OR "Education, Medical, Undergraduate"[Mesh] OR Health Professions Students[Title/Abstract] OR Health science students[Title/Abstract] OR nutrition students[Title/Abstract] OR nutritional science students[Title/Abstract] OR dietetic students[Title/Abstract] OR nursing students[Title/Abstract] OR Nutrition Major[Title/Abstract]) AND ("Weight Prejudice"[Mesh] OR ("Attitude of Health Personnel"[Mesh] AND "Body Weight"[Mesh]) OR ("Body Weight"[Mesh] AND stigma[title/abstract]) OR ("Body Weight"[Mesh] AND bias[title/abstract]) OR Weight bias[Title/Abstract] OR weight stigma[Title/Abstract] OR weight shame[Title/Abstract] OR weight guilt[Title/Abstract] OR weight prejudice[Title/Abstract] OR Weight Based Discrimination[Title/Abstract] OR Weight Discrimination[Title/Abstract] OR weight related bias[Title/Abstract] OR weight stereotypes[Title/Abstract] OR Weight-Based Attitudes[Title/Abstract]) |
| **Web of Science** | (TS=(Health Professions Students OR Health science students OR nutrition students OR nutritional science students OR dietetic students OR nursing students OR Nutrition Major OR medical students OR premedical students OR kinesiology students OR pharmacy students)) AND TS=(“Weight bias” OR “weight stigma” OR “weight shame” OR “weight guilt” OR “weight prejudice” OR “Weight Based Discrimination” OR “Weight Discrimination” OR “obesity stigma” OR “obesity bias” OR “weight related bias” OR “weight stereotypes” OR “Weight-Based Attitudes” OR “fat bias” OR “fat stigma” OR (weight AND “implicit bias”) OR (weight AND “explicit bias”) OR “weight stigmatization” )  **Limit – Articles, Review Articles, Early Access** |
