# Supplementary material for: Weight bias among undergraduate women with health-related majors: a systematic review
Source: J Eat Disord. 2025 Jun 12;13:108. doi: 10.1186/s40337-025-01275-1 (PMC12160116; doi:10.1186/s40337-025-01275-1)
Supplement: Supplementary file 2 — Supplementary Material 2. [file 40337_2025_1275_MOESM2_ESM.docx]

**Supplementary Table 2**. Joanna Briggs Institute Analytical Cross-Sectional Critical Appraisal Decisions by Item

| Study Author (Year) | 1^a^ | 2 | 3 | 4 | 5 | 6 | 7 | 8 | Overall Appraisal^b^ |
| --- | --- | --- | --- | --- | --- | --- | --- | --- | --- |
| Alameda 2015 | Yes | Yes | n/a | n/a | n/a | n/a | Yes | Yes | Include |
| Alvarenga 2022 | Yes | Yes | n/a | n/a | n/a | n/a | Yes | Yes | Include |
| Chambliss 2004 | Yes | Yes | n/a | n/a | n/a | n/a | Unclear | Yes | Include |
| Langdon 2016 | Yes | Yes | n/a | n/a | n/a | n/a | Yes | Yes | Include |
| Darling 2019 | Yes | Yes | n/a | n/a | n/a | n/a | Yes | Yes | Include |
| Ciao 2011 | Yes | Yes | n/a | n/a | n/a | n/a | Yes | Yes | Include |
| Ozydin 2022 | Yes | Yes | n/a | n/a | n/a | n/a | Yes | Yes | Include |
| Usta 2021 | Yes | Yes | n/a | n/a | n/a | n/a | Yes | Yes | Include |
| Yildiz 2019 | Yes | Yes | n/a | n/a | n/a | n/a | Yes | Yes | Include |
| Poon 2008 | Yes | Yes | n/a | n/a | n/a | n/a | Yes | Yes | Include |
| Tsai 2019 | Yes | Yes | n/a | n/a | n/a | n/a | Yes | Yes | Include |
| Rodriguez-Gazquez 2020 | Yes | Yes | n/a | n/a | n/a | n/a | Yes | Yes | Include |

**^a^ =** The Joanna Briggs Institute Critical Appraisal for Analytical Cross-Sectional studies contains 8 items with answer options of “Yes”, “ No”, “Unclear”, or “Not applicable”

^b^ = Overall Appraisal response options are “Include”, “Exclude”, or “Seek further info”

1 = Were the criteria for inclusion in the sample clearly defined?

2 = Were the study subjects and the setting described in detail?

3 = Was the exposure measured in a valid and reliable way?

4 = Were objective, standard criteria used for measurement of the condition?

5 = Were confounding factors identified?

6 = Were strategies to deal with confounding factors stated?

7 = Were the outcomes measured in a valid and reliable way?

8 = Was appropriate statistical analysis used?

JBI Qualitative Appraisal Checklist Source (Analytical Cross-Sectional Studies): Moola S, Munn Z, Tufanaru C, Aromataris E, Sears K, Sfetcu R, Currie M, Qureshi R, Mattis P, Lisy K, Mu P-F. Chapter 7: Systematic reviews of etiology and risk . In: Aromataris E, Munn Z (Editors). JBI Manual for Evidence Synthesis. JBI, 2020. Available from <https://synthesismanual.jbi.global>

**Supplementary Table 3**. Joanna Briggs Institute Qualitative Research Critical Appraisal Decisions by Item

| Study Author (Year) | 1^a^ | 2 | 3 | 4 | 5 | 6 | 7 | 8 | 9 | 10 | Overall Appraisal^b^ |
| --- | --- | --- | --- | --- | --- | --- | --- | --- | --- | --- | --- |
| Bessey | Unclear | Yes | Yes | Yes | Yes | No | Yes | Yes | Yes | Yes | Include |
| Dwyer | Unclear | Yes | Yes | Yes | Yes | No | No | Yes | Yes | Yes | Include |

**^a^ =** The Joanna Briggs Institute Critical Appraisal for Qualitative Research contains 10 items with answer options of “Yes”, “ No”, “Unclear”, or “Not applicable”

^b^ = Overall Appraisal response options are “Include”, “Exclude”, or “Seek further info”

1 = Is there congruity between the stated philosophical perspective and the research methodology?

2 = Is there congruity between the research methodology and the research question or objectives?

3 = Is there congruity between the research methodology and the methods used to collect data?

4 = Is there congruity between the research methodology and the representation and analysis of data?

5 = Is there congruity between the research methodology and the interpretation of results?

6 = Is there a statement locating the researcher culturally or theoretically?

7 = Is the influence of the researcher on the research, and vice- versa, addressed?

8 = Are participants, and their voices, adequately represented?

9 = Is the research ethical according to current criteria or, for recent studies, and is there evidence of ethical approval by an appropriate body?
10 = Do the conclusions drawn in the research report flow from the analysis, or interpretation, of the data?

JBI Qualitative Appraisal Checklist Source (Qualitative Studies): Lockwood C, Munn Z, Porritt K. Qualitative research synthesis: methodological guidance for systematic reviewers utilizing meta-aggregation. *Int J Evid Based Healthc*.2015;13(3):179–187.
